# Clustering time series based on dependence structure

**DOI:** 10.1371/journal.pone.0206753

**Published:** 2018-11-12

**Authors:** Beibei Zhang, Baiguo An

**Affiliations:** School of Statistics, Capital University of Economics and Business, Beijing, China; Feng Chia University, TAIWAN

## Abstract

The clustering of time series has attracted growing research interest in recent years. The most popular clustering methods assume that the time series are only linearly dependent but this assumption usually fails in practice. To overcome this limitation, in this paper, we study clustering methods applicable to time series with a general and dependent structure. We propose a copula-based distance to measure dissimilarity among time series and consider an estimator for it, where the strong consistency of the estimator is guaranteed. Once the pairwise distance matrix for time series has been obtained, we apply a hierarchical clustering algorithm to cluster the time series and ensure its consistency. Numerical studies, including a large number of simulations and analysis of practical data, show that our method performs well.

## Introduction

Massive amounts of data relating to time series are frequently collected in fields ranging from science, engineering, and business to economics, healthcare, and government. It is often desirable to find groups of similar time series in a set or panel of such series using clustering techniques. In the case of panel time series, especially for short time series, it is beneficial for model estimation and forecasting performance to pool time series with similar data-generating mechanisms [1]. Consequently, as one of the most frequently used exploratory techniques in data mining, clustering is a crucial step in time series analysis.

A popular approach to clustering time series is model-based clustering. This approach assumes that each time series is generated by a specific underlying model or a mixture of underlying probability distributions. The time series are considered similar when the models characterizing them are similar. The most commonly considered models include the ARIMA process [2], the GARCH model [3], and the dynamic regression model [1]. Researchers in machine learning have also used Markov chains [4] and hidden Markov models [5].

Unlike model-based clustering methods, distance-based methods cluster time series in a simple and efficient way, where the choice of a proper distance or dissimilarity measure is a critical step. Once the dissimilarity measure is determined, an initial pairwise dissimilarity matrix can be obtained and many conventional clustering algorithms can then be used to form groups of objects. Different distances are pursued according to the aim of time series clustering. The selected distance should be able to capture particular discrepancies between series that are relevant to the final objective of the clustering. The R package TSclust [6] provides a brief overview of well-established peer-reviewed time series dissimilarity measures, including measures based on raw data, extracted features, underlying parametric models, levels of complexity, and forecast behaviors. An interesting overview of time series clustering methods and their applications can be found in [7].

A central issue in the analysis of time series data is to consider the structure of the temporal dependence. It is often helpful for model estimation to cluster time series into several groups according to their underlying dependency structures. In most research on the issue, it is assumed that the temporal dependences of time series are only linear. The ARIMA model is the most commonly used linear model (see, for example, [2, 8–12]). However, the assumption of linearity often fails to hold in practice. When time series are nonlinearly dependent, linear methods suffer from a severe model mismatch problem. Scant attention has been paid in the literature thus far to the clustering of nonlinear time series. Nonparametric model-free methods are usually employed to deal with nonlinear problems. Dissimilarity in nonparametric distance-based clustering methods is measured by comparing serial features extracted from the original series that aim to represent the dynamic structure of each series, such as autocorrelation [13, 14], partial autocorrelation [15], cross-correlation [16], and spectral features [17]. Even though nonparametric methods do not make any model assumptions, most aforementioned dissimilarities are quantities related to Pearson’s correlation, which can only measure linear dependence. It is thus natural that these features are inadequate at recognizing more general, temporal, and nonlinear dependence structures. They are not expected to perform well at clustering more general time series, which was also shown in our simulation experiments in this study.

We ignore model-based clustering methods and focus on the distance-based nonlinear time series clustering approach due to its popularity and simplicity. In view of the aforementioned considerations, a distance measure of global dependence is required. Specifically, we need to construct a distance measure that can capture linear and nonlinear dependencies without requiring the specification of any kind of model. However, to the best of our knowledge, no prevalent measure of association or dependence can satisfy this requirement. Numerous diagnostics or tests can be used to only examine departures from independence, and include mutual information, entropy measure, and the Hellinger distance. They cannot distinguish between dependence structures or gauge dissimilarity between them.

In this paper, we propose a distance measure based on a copula function to measure the dissimilarity among the general serial dependence of two time series. The advantages of this distance measure can be summarized as follows: First, it overcomes the limitations of prevalent time series clustering methods designed for linear processes. Our simulations show that the proposed measure performs well, particularly in terms of classifying nonlinear models. As the proposed distance measure is designed in terms of a discrepancy in the serial structure of global dependence, which includes linear structures, it can also be used for linear processes. Second, it is nonparametric. To obtain the distance measure, we rely on an empirical estimator of the copula function. The superiority of the proposed estimation approach resides precisely in its ability to account for the divergence of global dependencies with no need to specify an exact model. Third, this is a rank-invariant approach, as copulas possess an invariance property with respect to a monotonically increasing transformation of the variables [18]. Fourth, we can theoretically guarantee the consistency of the distance estimator. Fifth, this distance measure takes a close form that can be efficiently computed.

The remainder of the paper is organized as follows: In Section 2, we described the proposed clustering method with relevant statistical properties. Simulations and analysis of data from a practical scenario are provided in Sections 3 and 4, respectively. A short conclusion is provided in Section 5. The proofs of all theorems are in the Appendix.

## Methodology

### Notations and copula-based distance

Let $X_{i}={\left(X_{i1},\cdots ,X_{iT_{i}}\right)}^{T}$ be the *i*-th time series (1 ≤ *i* ≤ *n*), where *T*_*i*_ is its length and *n* is the number of time series. We assume that these time series are strictly stationary drawn from *J*_0_ clusters, and the time series in each cluster share a common dynamic pattern. The purpose is to identify these *J*_0_ clusters. We propose a method based on the copula function to represent the dynamic pattern of the time series. Specifically, for a fixed positive integer *h*, (*X*_*ij*_, *X*_*i*(*j*+*h*)_) and (*X*_*i*′*j*_, *X*_*i*′(*j*+*h*)_) have the same copula function if time series *X*_*i*_ and *X*_*i*′_ belong to a common cluster.

For random variables *X* and *Y* with continuous marginal cumulative distribution functions *F*_*X*_(*x*) and *F*_*Y*_(*y*), respectively, we denote the joint cumulative distribution functions by *F*(*x*, *y*). Sklar’s theorem [19] claims that a unique copula function *C*(*u*, *v*) exists connecting *F*(*x*, *y*) to *F*_*X*_(*x*) and *F*_*Y*_(*y*) via *F*(*x*, *y*) = *C*(*F*_*X*_(*x*), *F*_*Y*_(*y*)), which is equivalent to $C\left(u,v\right)=F(F_{X}^{-1}\left(u\right),F_{Y}^{-1}\left(v\right))$. This means that the copula function *C*(*u*, *v*) can capture the structure of dependence between random variables *X* and *Y*. We use the copula to capture the dynamic pattern of the time series.

For each 1 ≤ *i* ≤ *n*, we denote by *C*_*i*,*h*_(*u*, *v*) the copula function of (*X*_*ij*_, *X*_*i*(*j*+*h*)_). For arbitrary *i* ≠ *i*′, we define the following copula-based Cramér-von Mises distance to measure the dissimilarity between time series *X*_*i*_ and *X*_*i*′_:

$$\begin{array}{c} D_{h}\left(i,i^{\prime }\right)=\sqrt{\int \int_{{\left[0,1\right]}^{2}}{\left(C_{i,h}\left(u,v\right)-C_{i^{\prime },h}\left(u,v\right)\right)}^{2}dudv}. \end{array}$$ (1)

The copula-based distance *D*_*h*_(*i*, *i*′) satisfies the following three classical properties:

1. (Nonnegativity) *D*_*h*_(*i*, *i*′) ≥ 0. Moreover, *D*_*h*_(*i*, *i*′) = 0 if and only if (*X*_*ij*_, *X*_*i*(*j*+*h*)_) and (*X*_*i*′*j*_, *X*_*i*′(*j*+*h*)_) share a common copula function.
2. (Symmetry) *D*_*h*_(*i*, *i*′) = *D*_*h*_(*i*′, *i*).
3. (Triangle inequality property) *D*_*h*_(*i*, *i*′) ≤ *D*_*h*_(*i*, *k*) + *D*_*h*_(*k*, *i*′).

Nonnegativity and symmetry are apparent in the foregoing, and the triangle inequality property can be obtained from the Cauchy–Schwarz inequality. Traditionally, the dependence structure of time series is often captured using an autocovariance-based linear correlation, which fails in nonlinear dependency structures. Nonlinear dependence may be caused by various nonlinear structures. The copula function does not make any assumptions about the model, and is a flexible method that can capture them. Moreover, copula-based distance is not affected by strict monotonic transformations (i.e., logarithmic transform and exponential transform) due to the property of mapping invariance of the copula function [18].

If time series *X*_*i*_(1 ≤ *i* ≤ *n*) are dependent in the order of one, we let *h* = 1 and directly use *D*_1_(*i*, *i*′) as the dissimilarity measure of *X*_*i*_ and *X*_*i*′_. If the times series are dependent in a higher order, the dissimilarity measure of *X*_*i*_, *X*_*i*′_ can be defined as the following weighted version:

$$\begin{array}{c} D\left(i,i^{\prime }\right)=\sum_{h=1}^{K}\omega_{h}D_{h}\left(i,i^{\prime }\right), \end{array}$$

where *K* is the highest order of dependence of time series *X*_*i*_ and *X*_*i*′_ considered in the dissimilarity measure definition, and *ω*_*h*_ is the weight of *D*_*h*_(*i*, *i*′). We can allow *ω*_*h*_ to decrease as *h* becomes larger.

The selection of *K* depends on the unknown underlying model. We may entertain several possible values of *K* and use model selection criteria such as AIC and BIC to select the optimal value of *K*. On this point, [20] claimed that the aim is not the goodness-of-fit to the underlying models but clustering them properly. We thus do not make any significant effort on this issue. In practice, the value of *K* should be chosen with specific knowledge of the application. It is often the case that the strongest serial dependency occurs in small lags, and a larger value of *K* means greater computational time and redundant information. Moreover, we can always obtain reasonably satisfactory results using small values of *K* in our simulations and applications. A large number of studies focusing on application have shown that it suffices for a large number of time series to use a lag of one to obtain goodness of fit. Therefore, in practice *K* = 1 is highly recommended.

### Estimation of copula-based distance

In practice, the copula function is usually unknown, and thus the copula-based distance (1) cannot be used directly. We propose a nonparametric estimation of the copula function that can be plugged into (1) to obtain the estimated distance.

Specifically, we denote the empirical distribution functions of (*X*_*it*_, *t* = 1, ⋯, *T*_*i*_) by ${\hat{F}}_{i}\left(x\right)=T_{i}^{-1}\sum_{t=1}^{T_{i}}I\left(X_{it}\leq x\right)$, where *I*(⋅) is an indicator function. For 1 ≤ *t* ≤ (*T*_*i*_ − *h*), we further define

$$\begin{array}{c} U_{it}=\frac{T_{i}}{T_{i}+1}{\hat{F}}_{i}\left(X_{it}\right),\;V_{it}=\frac{T_{i}}{T_{i}+1}{\hat{F}}_{i}\left(X_{i(t+h)}\right). \end{array}$$

Then, the nonparametric estimator for the copula function of (*X*_*it*_, *X*_*i*(*t*+*h*)_) (i.e., *C*_*i*, *h*_(*u*, *v*)) is defined as

$$\begin{array}{c} {\hat{C}}_{i,h}\left(u,v\right)=\frac{1}{T_{i}-h}\sum_{t=1}^{T_{i}-h}I\left(U_{it}\leq u\right)I\left(V_{it}\leq v\right). \end{array}$$

We replace *C*_*i*, *h*_(*u*, *v*) in (1) by ${\hat{C}}_{i,h}\left(u,v\right)$ and obtain the corresponding copula-based distance estimator:

$$\begin{array}{c} {\hat{D}}_{h}\left(i,i^{\prime }\right)=\sqrt{\int \int_{{\left[0,1\right]}^{2}}{\left({\hat{C}}_{i,h}\left(u,v\right)-{\hat{C}}_{i^{\prime },h}\left(u,v\right)\right)}^{2}dudv}. \end{array}$$ (2)

If we further assume that the time series are *α* mixing processes, ${\hat{C}}_{i,h}\left(u,v\right),{\hat{D}}_{h}\left(i,i^{\prime }\right)$ are strong consistency estimators for *C*_*i*,*h*_(*u*, *v*) and *D*_*h*_(*i*, *i*′), respectively. We summarize the results as the following theorem:

**Theorem 1** *Assume that time series X*_*i*_ = (*X*_*it*_, 1 ≤ *t* ≤ *T*_*i*_)(1 ≤ *i* ≤ *n*) *are strictly stationary α mixing processes*. *Then*, ${\hat{C}}_{i,h}\left(u,v\right)\overset{a.s.}{\to }C_{i,h}\left(u,v\right)$ *as T*_*i*_ → ∞ *and* ${\hat{D}}_{h}\left(i,i^{\prime }\right)\overset{a.s.}{\to }D_{h}\left(i,i^{\prime }\right)$, *as T*_*i*_, *T*_*i*′_ → ∞, *where* $\overset{a.s.}{\to }$ *denotes convergence with probability* 1.

The proof of Theorem 1 is provided in Appendix A. In practice, we need to calculate the value of ${\hat{D}}_{h}\left(i,i^{\prime }\right)$, which is easy. For the details of the calculation of ${\hat{D}}_{h}\left(i,i^{\prime }\right)$, see the following proposition:

**Proposition 1** *For i*, *i*′ = 1, ⋯, *n*, *define*

$$\begin{array}{c} L_{i,i^{\prime }}=\frac{\sum_{t=1}^{T_{i}-h}\sum_{t^{\prime }=1}^{T_{i^{\prime }}-h}\left(1-\max\left(U_{it},U_{i^{\prime }t^{\prime }}\right)\right)\left(1-\max\left(V_{it},V_{i^{\prime }t^{\prime }}\right)\right)}{\left(T_{i}-h\right)\left(T_{i^{\prime }}-h\right)}. \end{array}$$

*Then, for the copula-based distance estimator* ${\hat{D}}_{h}\left(i,i^{\prime }\right)$,

$$\begin{array}{c} {\hat{D}}_{h}\left(i,i^{\prime }\right)=\sqrt{L_{i,i}-2L_{i,i^{\prime }}+L_{i^{\prime },i^{\prime }}}. \end{array}$$ (3)

The proof of Proposition 1 is provided in Appendix B.

### Clustering algorithms based on copula distance

Once the pairwise distance between the time series has been obtained, we can apply any clustering method that uses a distance matrix as input, such as partition around medoids (PAM) [21], spectral clustering [22], and hierarchical clustering [23]. In PAM methods, we need to find a centroid time series in each cluster to represent the group. PAM methods require that the user specify the number of clusters. Through a hierarchical clustering algorithm, we can obtain a tree dendrogram built starting from the leaves and combining clusters to the trunk. The hierarchical clustering algorithm applied in this paper can be summarized as follows:

**Clustering Algorithm**
Step 1 For *i*, *i*′ = 1, ⋯, *n* and *i* ≠ *i*′, compute the estimator $\hat{D}\left(i,i^{\prime }\right)$ for the copula-based distance between time series pair *X*_*i*_ and *X*_*i*′_. Then, we can obtain the distance matrix. Treat each time series as its own cluster.
Step 2 For *J* = *n*, *n* − 1, ⋯, 2:
  a Compare all pairwise dissimilarity measures among the *J* clusters and identify the pair of clusters most similar. Merge them into as one.
  b Compute the pairwise dissimilarities of the new *J* − 1 clusters.

It is worth mentioning that dissimilarities should be defined among clusters based on distances between time series prior to applying the clustering algorithm. Single linkage, complete linkage, average linkage, centroid linkage, and Ward’s linkage are the most common methods to extend dissimilarities among observations to dissimilarities among clusters [24].

In practice, The computations of clustering can be performed in a more efficient way. [25] introduced a recurrence formula which can be used to compute the updated inter-cluster distance values efficiently. If a clustering procedure does not satisfy such a recurrence relation, the initial data should be retained throughout the entire process when updating cluster distances. All these aforementioned linkage methods fit into the Lance-Williams formalism, and can therefore easily be implemented with user-defined time series distance. In this paper, the efficient method of implementing clustering is to store the matrix of copula distances and update inter-cluster distance using Lance-Williams recursive formula.

Specifically, the traditional Ward’s linkage method minimizes the increase in total within-cluster sum of squared error. This increase is proportional to the squared Euclidean distance between cluster centers. Here, we extends the classical Ward’s linkage method that relies on Euclidean distance to copula distance, which still shares the same parameters in the update formula with original Ward’s linkage method [26]. With the Lance-Williams formula, we do not need to assign the cluster center to compute the new inter-cluster distance.

Recall that we assume these time series are drawn from *J*_0_ clusters. We denote these *J*_0_ clusters by $\left\{M_{1},\cdots ,M_{J_{0}}\right\}$. Let *C*_*j*0,*h*_(*u*, *v*) be the common copula shared by the time series in the *j*-th cluster. We further define

$$\begin{array}{c} D_{0,h}\left(j,j^{\prime }\right)=\sqrt{\int \int_{{\left[0,1\right]}^{2}}{\left(C_{j0,h}\left(u,v\right)-C_{j^{\prime }0,h}\left(u,v\right)\right)}^{2}dudv}, \end{array}$$

which is the copula-based distance between the *j*-th and the *j*′-th clusters. Let *ϵ* = min_*j*≠*j*′_ *D*_0,*h*_(*j*, *j*′), which is the minimal distance between the clusters. Then, for the foregoing clustering algorithm, the following theorem holds:

**Theorem 2** *Assume that time series X*_*i*_ = (*X*_*it*_, 1 ≤ *t* ≤ *T*_*i*_)(1 ≤ *i* ≤ *n*) *are strictly stationary α mixing processes and ϵ* > 0. *For J* > *J*_0_ *in Step 2 of Clustering Algorithm, the obtained J* − 1 *clusters are denoted by* $\left\{{\hat{M}}_{1},\cdots ,{\hat{M}}_{J-1}\right\}$. *Then, as* min_*i*_{*T*_*i*_}→∞, *with probability 1 we have that for every* ${\hat{M}}_{j}$ *with* 1 ≤ *j* ≤ *J* − 1, *there exists a j*′ *such that* ${\hat{M}}_{j}\subset M_{j^{\prime }}$.

We call the results of Theorem 2 the consistency of clustering. The proof of Theorem 2 is provided in Appendix C. If *J*_0_ is known, we can directly cluster the time series into *J*_0_ groups. However, the number of clusters *J*_0_ is usually unknown, and we should detect the optimal number of clusters in practice. A useful approach to determine this optimal number is the silhouette method [27]. For each time series *X*_*i*_ with *i* = 1, 2, …, *n*, its silhouette width *s*(*i*) is defined as

$$\begin{array}{c} s\left(i\right)=\frac{b(i)-a(i)}{max(a(i),b(i))}, \end{array}$$

where *a*(*i*) is the average copula distance between series *X*_*i*_ and all other time series of the cluster to which *X*_*i*_ belongs, and *b*(*i*) is the average copula distance between series *X*_*i*_ and time series in neighboring cluster, i.e., the nearest cluster to which it does not belong. Let the set of time series be partitioned into *J* clusters. The corresponding average silhouette width is defined as

$$\begin{array}{c} Sil\left(J\right)=\frac{\sum_{i=1}^{n}s\left(i\right)}{n}. \end{array}$$

The average silhouette of the clusters is calculated according to the number of clusters. A high average silhouette width indicates good clustering. Thus, the optimal number of clusters *J* is that which maximizes the average silhouette over a range of possible values for *J*. We choose the number of clusters as J*, which yields the maximum value of *Sil*(*J*).

## Simulations

We used three examples to assess the performance of nonlinear time series clustering based on our proposed distance measure. For the sake of comparison, in each simulation scenario, we performed clustering using some representative dissimilarity measures proposed in the literature, including model-based measures *d*_*PIC*_ of [8] and *d*_*M*_ of [28], which are based on ARIMA models. We also made comparisons with model-free dissimilarity measures. In the temporal domain, distances *d*_*ACF*_ and *d*_*PACF*_ are defined as Euclidean distances between the estimated ACF and PACF using a number of significant lags. In the frequency domain, the dissimilarity measures were designed to assess the discrepancy between corresponding spectral densities. [15] proposed distance measures based on the periodogram, Euclidean distances between periodograms (*d*_*P*_), log-periodograms (*d*_*LP*_), normalized periodograms (*d*_*NP*_), and log-normalized periodograms(*d*_*LNP*_). The other nonparametric dissimilarity measures in the frequency domain, *d*_*W*(*DLS*)_, *d*_*W*(*LK*)_, *d*_*GLK*_, and *d*_*ISD*_ proposed in [17], were also considered. They are different versions of nonparametric spectral dissimilarity measures. The differences among them were in terms of estimation methods of spectral density and discrepancy functions. For more details concerning these methods, the interested reader can refer to [6].

In the simulation experiments, the ground truth was known in advance. We assessed the clustering methods by using the cluster similarity index proposed in [29], which is defined as

$$\begin{array}{c} Sim\left(G,A\right)=\frac{1}{J_{0}}\sum_{i=1}^{J_{0}}\max_{1\leq j\leq J_{0}}Sim\left(G_{i},A_{j}\right), \end{array}$$

where $G=\{G_{1},G_{2},\cdots ,G_{J_{0}}\}$ are the set of *J*_0_ true clusters, $A=\{A_{1},A_{2},\cdots ,A_{J_{0}}\}$ is the solution to the clusters by the clustering method evaluated, and

$$\begin{array}{c} Sim\left(G_{i},A_{j}\right)=\frac{2|G_{i}\cap A_{j}|}{|G_{i}\left|+\right|A_{j}|}, \end{array}$$

where |⋅| denotes the cardinality of an arbitrary set. The similarity index has values ranging from zero to one, with one corresponding to the case when G and A are identical.

### Example 1: Nonlinear time series clustering

In this example, we considered the following four models:

1. Threshold autoregressive (TAR) model
  $$\begin{array}{c} X_{t}=0.5X_{t-1}I\left(X_{t-1}\leq 0\right)-2X_{t-1}I\left(X_{t-1}>0\right)+\varepsilon_{t}, \end{array}$$
2. Exponential autoregressive (EXPAR) model
  $$\begin{array}{c} X_{t}=\left(0.3-10\text{exp}\left\{-X_{t-1}^{2}\right\}\right)X_{t-1}+\varepsilon_{t}, \end{array}$$
3. Linear moving average (MA) model
  $$\begin{array}{c} X_{t}=\varepsilon_{t}-0.4\varepsilon_{t-1}, \end{array}$$
4. Nonlinear moving average (NLMA) model
  $$\begin{array}{c} X_{t}=\varepsilon_{t}-0.5\varepsilon_{t-1}+0.8\varepsilon_{t-1}^{2}, \end{array}$$

where the error process *ε*_*t*_ independently followed *N*(0, 1). These models were used in [30] for linearity tests and [17] to study time series clustering. Except for model (3), the others were conditional mean nonlinear models. The stationarity of these models can be guaranteed. We here only take the TAR model as an instance to illustrate its stationarity. For a TAR model

$$\begin{array}{c} X_{t}=\left(\gamma +\alpha X_{t-1}\right)I\left(X_{t-1}\leq r\right)+\left(\delta +\beta X_{t-1}\right)I\left(X_{t-1}>r\right)+\varepsilon_{t}, \end{array}$$

where {*ε*_*t*_} are independent and identically distributed with zero mean and a finite variance. Then the necessary and sufficient condition for the strictly stationarity to above TAR model when *γ* = *δ* = 0 is *α* < 1, *β* < 1 and *αβ* < 1 [31–34]. In model (1), we set *γ* = *δ* = *r* = 0, and *α* = 0.5, *β* = −2, hence the necessary and sufficient condition holds, and furhter the stationarity of the TAR model (1) is guaranteed.

**Fig 1:**
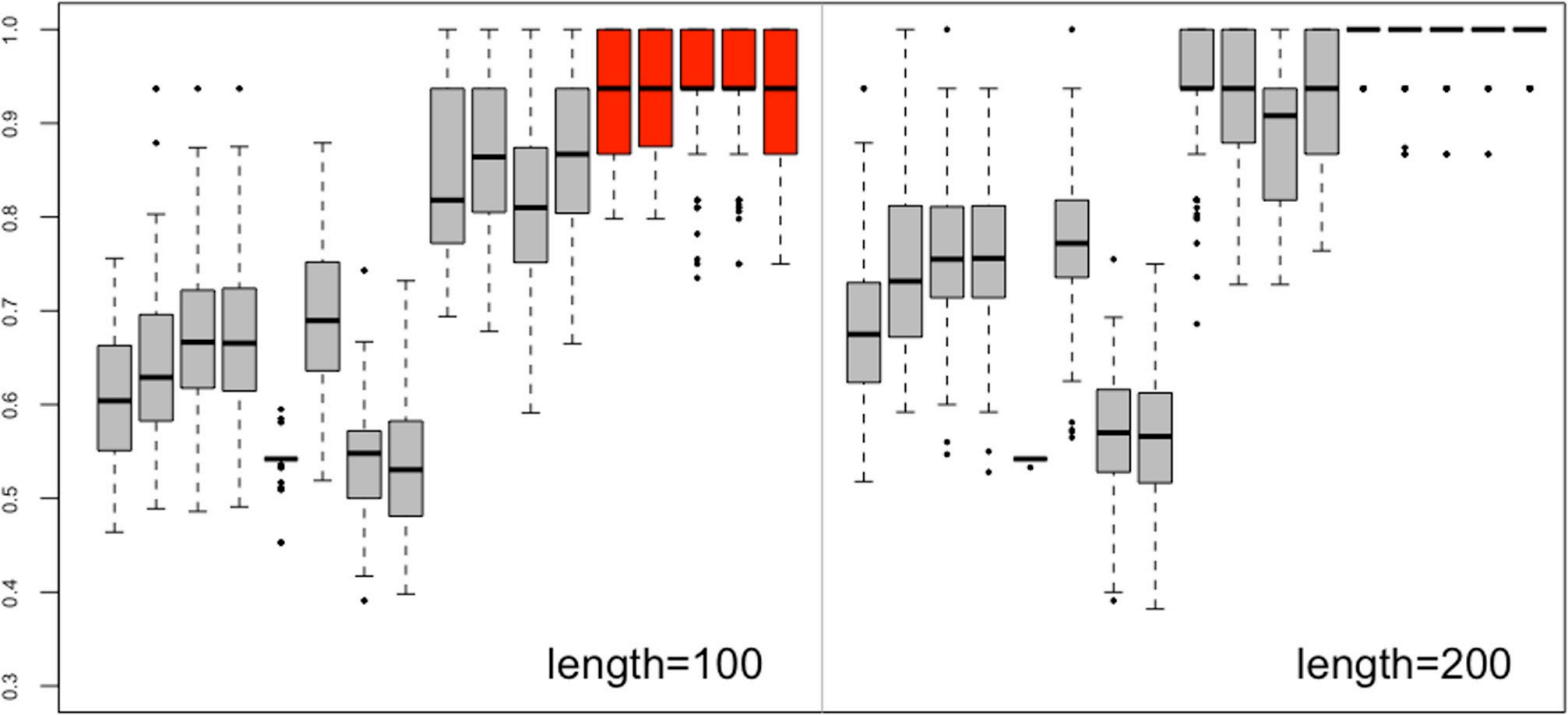
Example 1. Boxplot of clustering similarity indices. The distances from left to right: *d*_*ACF*_, *d*_*PACF*_, *d*_*PIC*_, *d*_*M*_, *d*_*P*_, *d*_*LP*_, *d*_*NP*_, *d*_*LNP*_, *d*_*DLS*_, *d*_*LK*_, *d*_*GLK*_, *d*_*ISD*_, *Copula*(*K* = 1), *Copula*(*K* = 2), *Copula*(*K* = 3), *Copula*(*K* = 4), and *Copula*(*K* = 5).

We generated four time series from each model, and thus the sample size was 16. We set the lengths of all series as a common parameter *T*, and we considered two values of *T* (i.e. *T* = 100, 200). For sake of simplicity, in all of the experiments we used uniform weight for copula distance, that is *w*_*h*_ = 1 for 1 ≤ *h* ≤ *K*. The experiment was repeated 100 times using all the aforementioned distance measures. The clustering similarity indices were calculated and summarized by the boxplot in Fig 1. The distance based on the copula function always yielded the best performance. For each of the distances, a larger series size seems to benefit more. When the series length *T* = 200, the similarity indices of the copula distance were almost equal to one, which meant that the copula distance could cluster the series into the true group from which they were generated. The first eight distances (*d*_*ACF*_ ∼ *d*_*LNP*_) needed the assumption of linearity of the time series. Therefore, their results were significantly worse than those of the other nonparametric distances (*d*_*DLS*_ ∼ *d*_*ISD*_) in this example.

**Fig 2:**
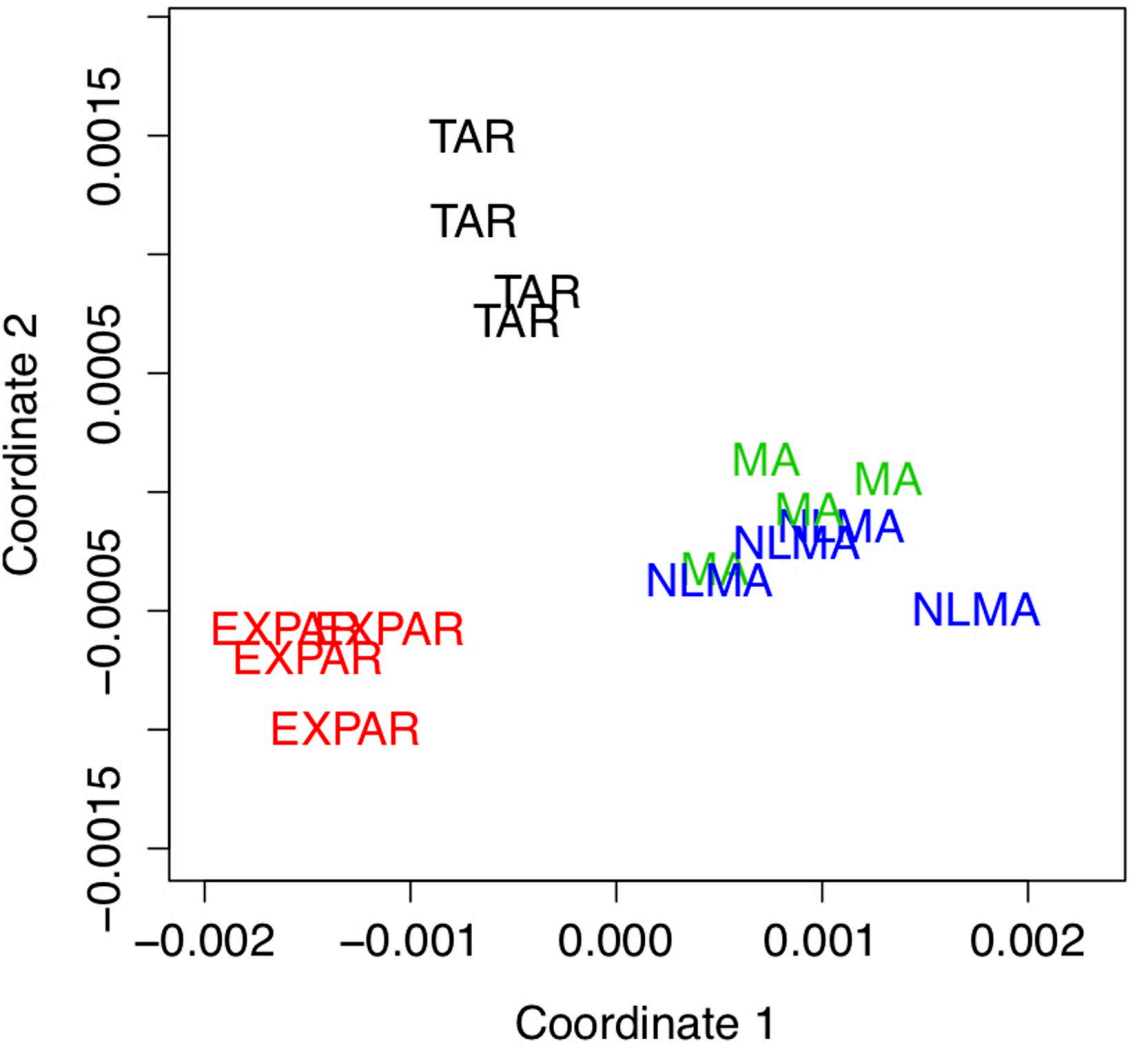
Example 1. Multidimensional scaling plot.

Fig 2 shows the multidimensional scaling (MDS) plot [35] used to visualize observations in two dimensions (*T* = 200), where the dissimilarity among the time series was based on our proposed copula distance. With the dissimilarity measures obtained from the data, the MDS plot sought a lower-dimensional representation of the data that preserved pairwise distances as closely as possible. Fig 2 shows a clear separation of the four clusters and the capability of copula distance to discriminate among them. Furthermore, it is evident that the series from the MA models and NLMA models are closer to one another because both models expressed time series as a function of white noise.

To gain further insights into copula distance, based on Example 1, we designed two more challenging clustering tasks. The first involved increasing heterogeneity within each cluster and the second explored the performance of copula distance when the strength of nonlinear dependence was changing.

### Example 2: Nonlinear time series clustering with increased intra-cluster heterogeneity

In this example, we considered models similar to those in Example 1. The length of each time series was set to *T* = 200 here. To enhance the difficulty of nonlinear time series clustering, we made some changes to each model. For model (1), TAR model is generalized to the Smooth Transition Autoregressive (STAR) model, which allows for higher degree of flexibility in model parameters. Similarly, for model (3), linear MA model was replaced by the Smooth Transition Moving Average (STMA) model. For models (2) and (4), instead of using a fixed constant in each model, we used varying coefficients generated randomly from some given uniform distributions. Specifically, the models considered were:

1. Smooth transition autoregressive (STAR) model
  $$\begin{array}{c} X_{t}=0.5X_{t-1}-2.5X_{t-1}F\left(X_{t-1}\right)+\varepsilon_{t}, \end{array}$$
  where *F*(*X*_*t*−1_) = (1 + exp(−*X*_*t*−1_))^−1^ is the smooth transition function.
2. Exponential autoregressive (EXPAR) model
  $$\begin{array}{c} X_{t}=\left(a_{2}-b_{2}\text{exp}\left\{-X_{t-1}^{2}\right\}\right)X_{t-1}+\varepsilon_{t},a_{2}∼U\left(0.2,0.6\right),b_{2}∼U\left(6,12\right); \end{array}$$
3. Smooth transition moving average (STMA) model
  $$\begin{array}{c} X_{t}=\varepsilon_{t}-0.4\varepsilon_{t-1}+0.8\varepsilon_{t-1}F\left(X_{t-1}\right)+\varepsilon_{t}, \end{array}$$
  where the smooth transition function is specified as
  $$\begin{array}{c} F\left(X_{t-1}\right)=1-\exp\left(-X_{t-1}^{2}\right). \end{array}$$
4. Nonlinear moving average (NLMA) model
  $$\begin{array}{c} X_{t}=\varepsilon_{t}-a_{4}\varepsilon_{t-1}+b_{4}\varepsilon_{t-1}^{2},a_{4}∼U\left(0.3,0.7\right),b_{4}∼U\left(0.2,0.9\right). \end{array}$$

**Fig 3:**
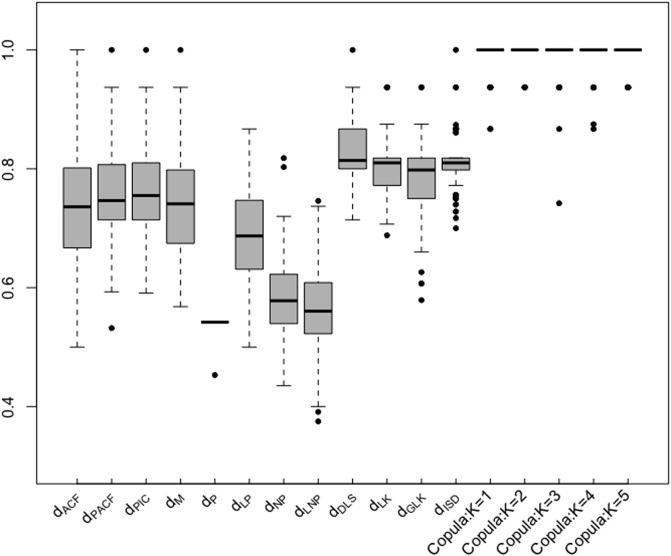
Example 2. Boxplot of clustering similarity indices: heterogeneity enlarged.

The results are shown in Fig 3. As the heterogeneity in each group increased, almost all of the distance measured led to different levels of performance degradation except copula distance. The copula distance still generated the best performance among all measures, and its performance has hardly any degradation.

### Example 3: Nonlinear time series clustering by adjusting nonlinear strength

In this example, we studied the sensitivity of copula distance to the nonlinear strength of the time series with the length *T* = 200. We wanted to determine the clustering performance of copula distance when the nonlinear strength of the time series varied. We controlled the strength of nonlinearity by adjusting the coefficients of the models considered in Example 1 as follows:

1. Threshold autoregressive (TAR) model
  $$\begin{array}{c} X_{t}=0.5X_{t-1}I\left(X_{t-1}\leq 0\right)-b_{1}X_{t-1}I\left(X_{t-1}>0\right)+\varepsilon_{t}; \end{array}$$
2. Exponential autoregressive (EXPAR) model
  $$\begin{array}{c} X_{t}=\left(0.3-b_{2}\text{exp}\left\{-X_{t-1}^{2}\right\}\right)X_{t-1}+\varepsilon_{t}; \end{array}$$
3. Linear moving average (MA) model
  $$\begin{array}{c} X_{t}=\varepsilon_{t}-0.4\varepsilon_{t-1}; \end{array}$$
4. Nonlinear moving average (NLMA) model
  $$\begin{array}{c} X_{t}=\varepsilon_{t}-0.5\varepsilon_{t-1}+b_{4}\varepsilon_{t-1}^{2}. \end{array}$$

We can see that model (3) remains identical to that in Example 1 and, if (*b*_1_, *b*_2_, *b*_4_) was equal to (2, 10, 0.8), the remaining three models were identical to those in Example 1. On the contrary, if we set (*b*_1_, *b*_2_, *b*_4_) equal to (−0.5, 0, 0), the models degenerated to the following linear models:

1. *X*_*t*_ = 0.5*X*_*t*−1_ + *ε*_*t*_;
2. *X*_*t*_ = 0.3*X*_*t*−1_ + *ε*_*t*_;
3. *X*_*t*_ = *ε*_*t*_ − 0.4*ε*_*t*−1_;
4. *X*_*t*_ = *ε*_*t*_ − 0.5*ε*_*t*−1_.

**Fig 4:**
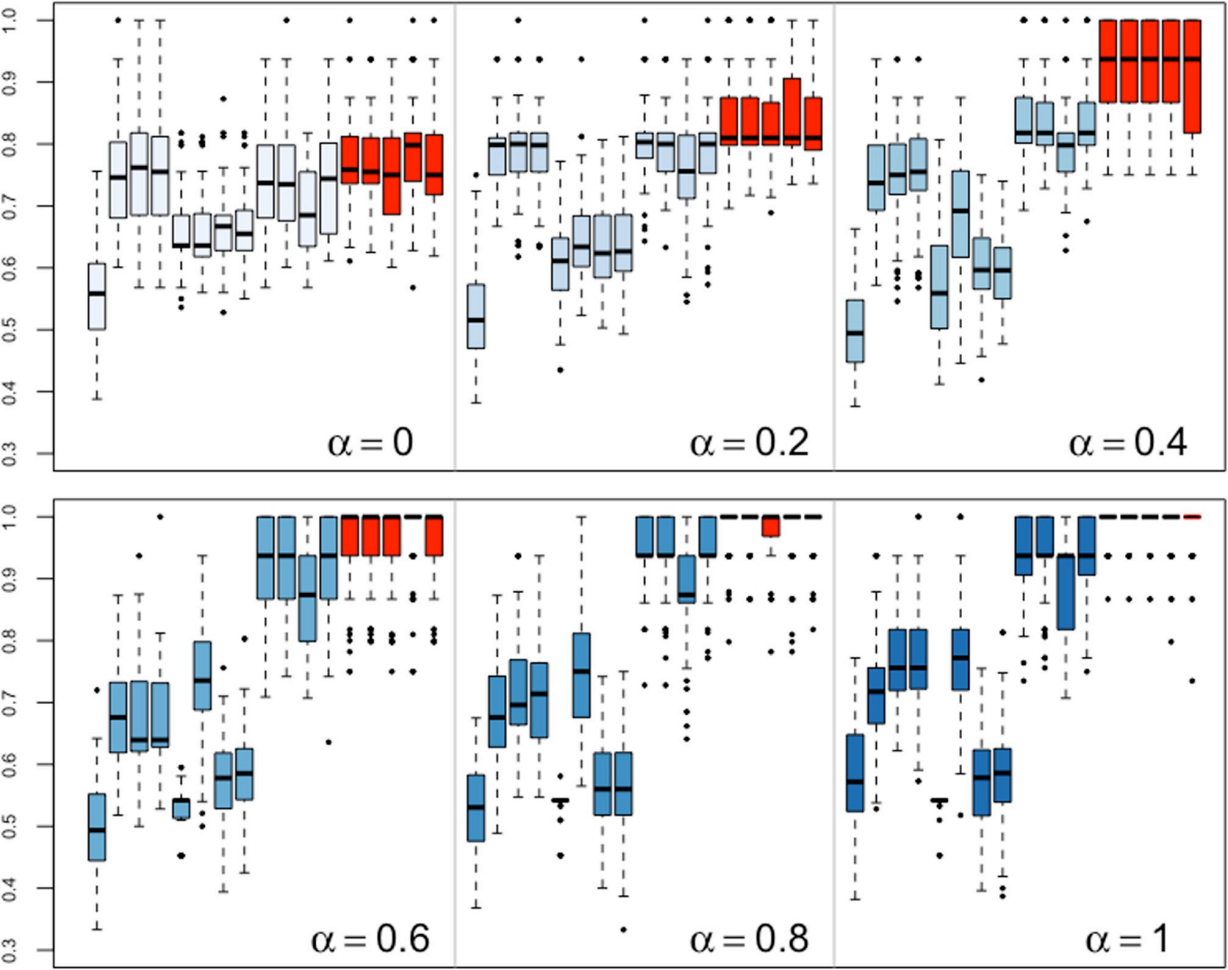
Example 3. Boxplots of clustering similarity indices: adjusting nonlinear strength. The distances from left to right: *d*_*ACF*_, *d*_*PACF*_, *d*_*PIC*_, *d*_*M*_, *d*_*P*_, *d*_*LP*_, *d*_*NP*_, *d*_*LNP*_, *d*_*DLS*_, *d*_*LK*_, *d*_*GLK*_, *d*_*ISD*_, *Copula*(*K* = 1), *Copula*(*K* = 2), *Copula*(*K* = 3), *Copula*(*K* = 4), and *Copula*(*K* = 5).

We can see that the strength of nonlinear dependency decreased when (*b*_1_, *b*_2_, *b*_4_) changed from (2, 10, 0.8) to (−0.5, 0, 0). Therefore, if we assigned (*b*_1_, *b*_2_, *b*_4_) = (2.5*α* − 0.5, 10*α*, 0.8*α*), 0 ≤ *α* ≤ 1, the size of *α* represented the strength of nonlinear dependence. The larger the value of *α*, the stronger the nonlinear dependence. When *α* = 1, this strength was the highest, which is the situation in Example 1. On the contrary, when *α* = 0 the strength of nonlinear dependence was the weakest, which was linear. We provided six uniformly spaced values in [0, 1] to *α* (i.e., *α* = 0, 0.2, 0.4, 0.6, 0.8, 1), and the results of clustering are shown in Fig 4 below.

From Fig 4, we see that when the time series were simulated from linear models, i.e., *α* = 0, the distance measures based on the assumption of model linearity yielded the best performance, such as *d*_*PACF*_, *d*_*PIC*_, and *d*_*M*_. The performance of copula distance was not inferior to that of the other methods. As nonlinear strength increased (i.e., *α* increased), the advantage of nonparametric distance measures become ever more apparent while the clustering performance of methods based on linearity degenerated. In most cases, copula distance yielded far better results than the competition.

## Real data analysis

We further illustrate the use of copula distance for time series clustering with two practical examples.

### Case 1: Annual real GDP data analysis

**Fig 5:**
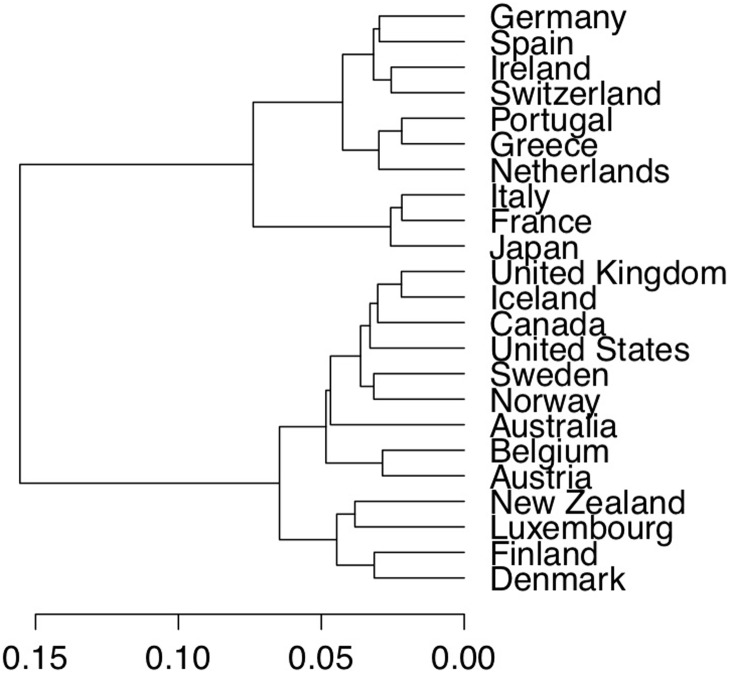
Annual real GDP data analysis. GDP clustering dendrogram based on copula distance with *K* = 2.

We considered data concerning the gross domestic product (GDP) obtained from https://www.conference-board.org/retrievefile.cfm?filename=Output-Labor-and-Labor-Productivity-1950-20111.xls&type=subsite. It contained the annual real GDP of the 23 most developed countries in the world from 1950 to 2011: Austria, Belgium, Denmark, Finland, France, Germany, Greece, Iceland, Ireland, Italy, Luxembourg, the Netherlands, Norway, Portugal, Spain, Sweden, Switzerland, United Kingdom, Canada, the United States, Australia, New Zealand, and Japan. We considered data normalized by the EKS method [36]. We used annual GDP growth rate *log*(*GDP*_*t*_) − *log*(*GDP*_*t*−1_) in the clustering procedures rather than annual GDP. These series were clustered via Ward’s linkage method based on copula distance. When *K* = 2, the dendrogram is shown in Fig 5. The result was relatively insensitive to the maximum lag *K* used, with similar clustering results obtained when *K* ranged from two to nine.

**Fig 6:**
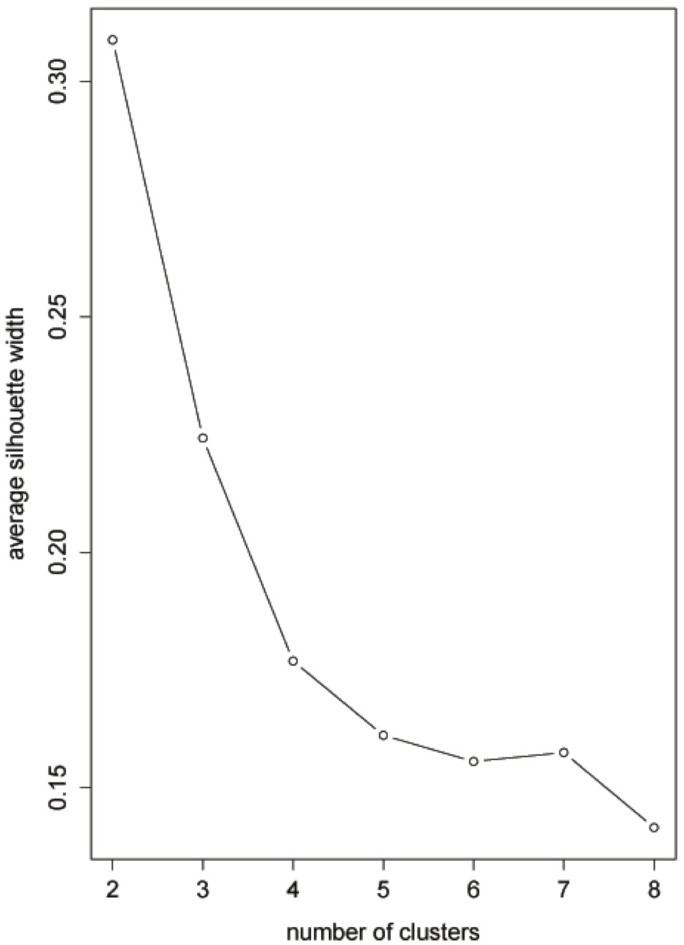
Annual real GDP data analysis. Plot of average silhouette coefficient (*K* = 2).

In practice, we do not know how many distinct populations generate *n* time series. In general, as in any cluster analysis, the optimal number of clusters can be chosen according to some objective criterion, such as the average silhouette criterion. The average silhouette of the data is a useful criterion for assessing the natural number of clusters, which can be determined by maximizing the coefficient. It is a measure of how tightly grouped all data in the cluster are. The average silhouette coefficients were examined for different numbers of clusters, and two clusters appeared to yield a compact solution (Fig 6).

**Fig 7:**
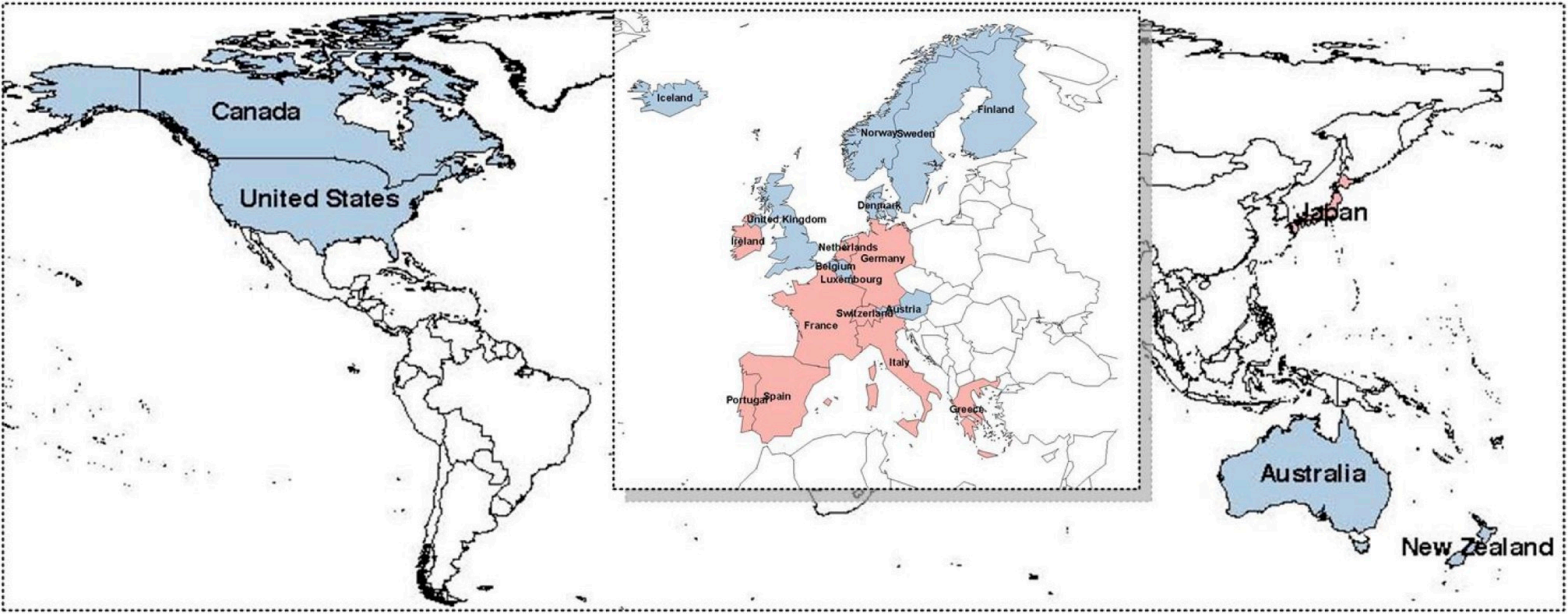
Annual real GDP data analysis. Two group of GDP data in map based on copula distance (*K* = 2).

Fig 7 shows the grouping of the two clusters. In this figure, the two colors represent two groups. To display the clustering effect of copula distance, the area occupied by Europe is magnified many times. It is interesting to note that the countries were grouped primarily by geographical location. The group in blue contains five northern European countries and nearly all developed non-European countries except Japan. The northern European countries have a long history of cooperation and so have much in common. The second group is in red, and includes central European countries, southern European countries, and western European countries to the south.

### Case 2: Population growth data analysis

**Fig 8:**
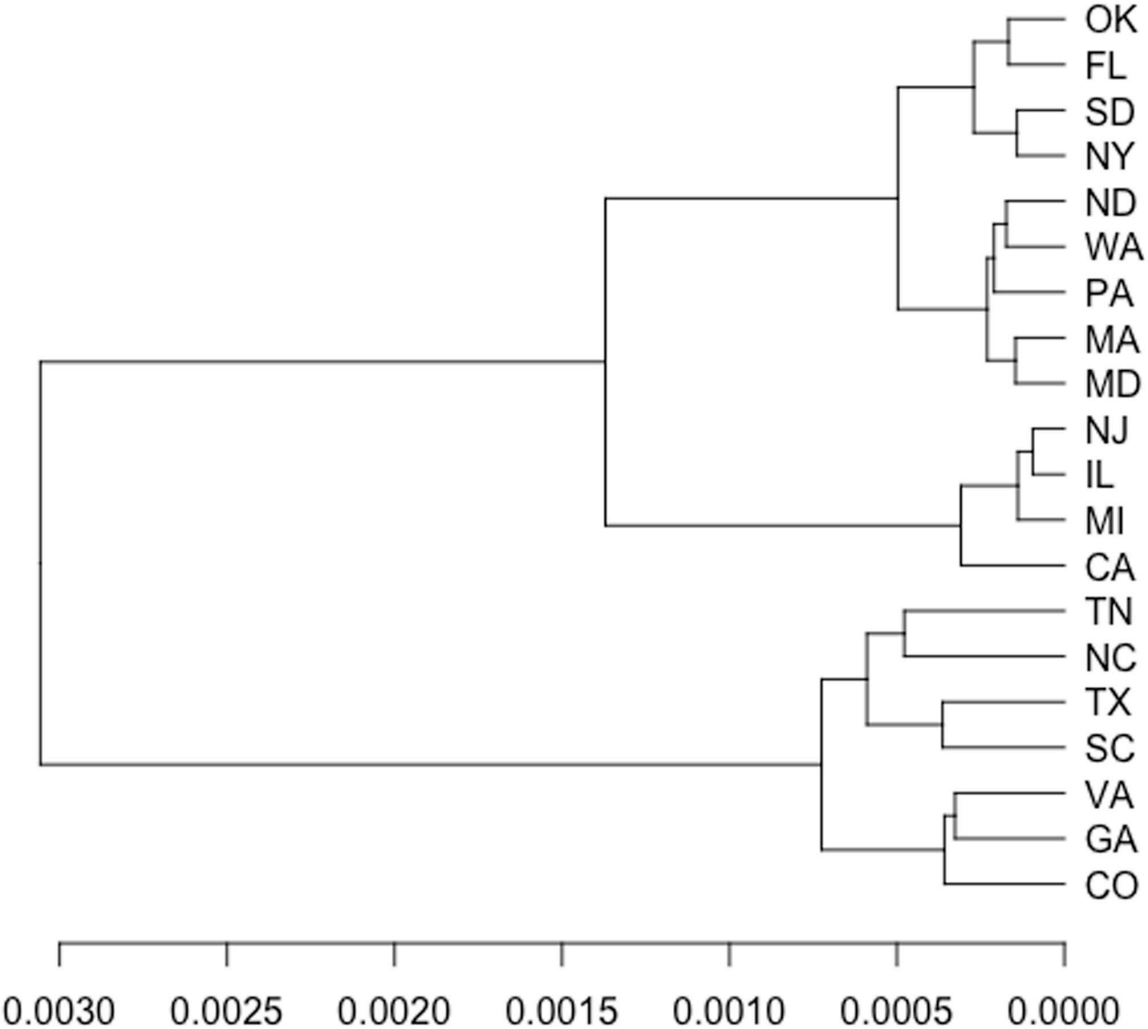
Population growth data analysis. Population Growth clustering dendrogram based on copula distance with *K* = 2.

**Fig 9:**
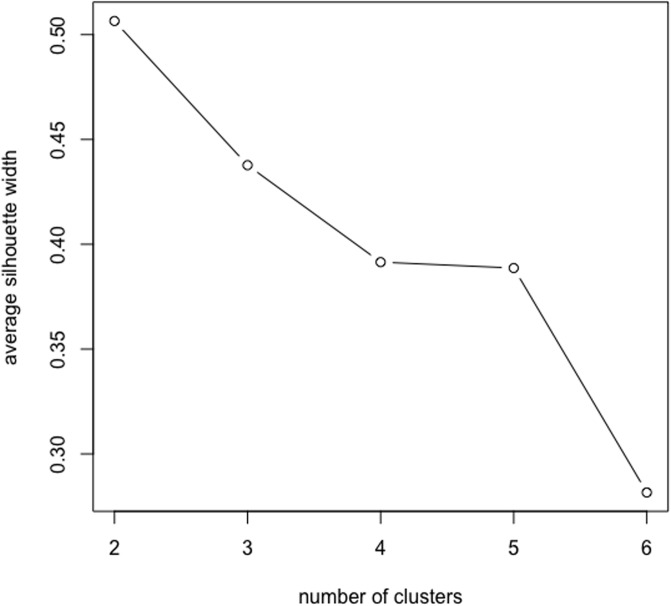
Population growth data analysis. Plot of average silhouette coefficient (*K* = 2).

This application considers the annual population series of 20 US states, and is aimed at identifying similarities among the population growth trend. This data is obtained from the U.S Census Bureau, Population Distribution Division (https://www.census.gov/programs-surveys/popest/data/data-sets.2007.html). It is a collection of time series of the population estimates from 1991 to 2010 in 20 states of the US, and has been used in [10]. In [10], two different groups of time series in the dataset were identified. Group 1 consisting of CA, CO, FL, GA, MD, NC, SC, TN, TX, VA, and WA had an exponentially growth trend, while Group 2 consisting of IL, MA, MI, NJ, NY, OK, PA, ND, and SD had a stable trend. In this case, we assume the above finding is the ground truth. In the following analysis, we use the growth series by taking log difference of the original time series. We cluster the data with hierarchical clustering algorithm using Ward’s linkage method. The dendrogram clustered by copula distance with Ward’s linkage method are shown in Fig 8. As we know, in this application the optimal number of clusters is 2. We can get the exact same optimal cluster number by maximizing the average silhouette width (see Fig 9). The copula distance method has the similarity index 0.8. If we section the dendrogram at the highest level we can obtain two groups, and all of the states are correctly classified except three states, CA, FL and MD.

## Conclusion

The ability to successfully cluster sets of time series is a popular area of research in many fields. In this paper, we proposed a clustering method that is not limited to linear processes. Because of the diversity of the structures of dependence of time series, we avoided model-based clustering and used the distance-based method instead. We proposed a distance measure based on the copula function to measure the dissimilarity between the general serial dependence of time series. This distance can be calculated easily by the empirical estimator of the copula function. We theoretically guaranteed the consistency of the distance estimator as well. This method takes advantage of the ability of the copula function to measure the global dependence of the time series and fills gaps in research on time series clustering. Three simulation examples and the examination of a practical scenario illustrated the usefulness of our method. However, our proposed method has its limitation. Beyond the linear time series, there are infinitely many nonlinear forms to be explored. In this paper we only explore nonlinear relationship between lagged variables, which is only one general kind of nonlinear time series.

## Appendixes

### Appendix A: Proof of Theorem 1

For every 1 ≤ *i* ≤ *n*, denote by *F*_*i*_(*x*) the marginal distribution function of *X*_*it*_. Then we will prove the theorem by the following four steps.

Step 1 For arbitrary *u*, *v* ∈ [0, 1], define
  $$\begin{array}{c} {\tilde{C}}_{i,h}\left(u,v\right)={\left(T_{i}-h\right)}^{-1}\sum_{t=1}^{T_{i}-h}I\left(F_{i}\left(X_{it}\right)\leq u,F_{i}\left(X_{i(t+h)}\right)\leq v\right). \end{array}$$
  In this step, we will prove that as *T*_*i*_ → ∞, ${\tilde{C}}_{i,h}\left(u,v\right)\overset{a.s.}{\to }C_{i,h}\left(u,v\right)$.
  Specifically, for 1 ≤ *i* ≤ *n* and 1 ≤ *t* ≤ *T*_*i*_ − *h*, let *Y*_*it*_ = *I*(*F*_*i*_(*X*_*it*_) ≤ *u*, *F*_*i*_(*X*_*i*(*t*+*h*)_) ≤ *v*). Because *X*_*i*_ = (*X*_*it*_, 1 ≤ *t* ≤ *T*_*i*_) is a strictly stationary *α* mixing processe, one can see that *Y*_*i*_ = {*Y*_*it*_, 1 ≤ *t* ≤ *T*_*i*_} is also a strictly stationary *α* mixing processe. Then by Proposition 2.8 of [37], one can see that as *T*_*i*_ → ∞,
  $$\begin{array}{c} {\tilde{C}}_{i,h}\left(u,v\right)\overset{a.s.}{⟶}P\left(F_{i}\left(X_{it}\right)\leq u,F_{i}\left(X_{i(t+h)}\right)\leq v\right)=C_{i,h}\left(u,v\right). \end{array}$$
Step 2 In this step, we will prove that as *T*_*i*_ → ∞,
  $$\begin{array}{c} \underset{-\infty <x<\infty }{\sup}\left|{\hat{F}}_{i}\left(x\right)-F_{i}\left(x\right)\right|\overset{a.s.}{⟶}0. \end{array}$$
  Because *X*_*i*_ = (*X*_*it*_, 1 ≤ *t* ≤ *T*_*i*_) is a strictly stationary *α* mixing processe, *X*_*i*_ is ergodic. Then by the theorem of [38], the Glivenko-Cantelli theorem holds for time series *X*_*i*_, hence we have that as *T*_*i*_ → ∞,
  $$\begin{array}{c} \underset{-\infty <x<\infty }{\sup}\left|{\hat{F}}_{i}\left(x\right)-F_{i}\left(x\right)\right|\overset{a.s.}{⟶}0. \end{array}$$
Step 3 In this step, we will prove that ${\hat{C}}_{i,h}\left(u,v\right)\overset{a.s.}{\to }C_{i,h}\left(u,v\right)$. Define
  $$\begin{array}{c} A_{i}=\{\lim_{T_{i}\to \infty }\underset{-\infty <x<\infty }{\sup}|{\hat{F}}_{i}\left(x\right)-F_{i}\left(x\right)\left|=0\right\}. \end{array}$$
  By the result of Step 2, we know that *P*(*A*_*i*_) = 1. Consequently, it suffices to prove ${\hat{C}}_{i,h}\left(u,v\right)\overset{a.s.}{\to }C_{i,h}\left(u,v\right)$ on the event *A*_*i*_.
  Specifically,
  $$\begin{array}{ccc} {\hat{C}}_{i,h}\left(u,v\right) & = & \frac{1}{T_{i}-h}\sum_{t=1}^{T_{i}-h}I\left(U_{it}\leq u\right)I\left(V_{it}\leq v\right)\\ & = & \frac{1}{T_{i}-h}\sum_{t=1}^{T_{i}-h}I(\frac{T_{i}}{T_{i}+1}{\hat{F}}_{i}\left(X_{it}\right)\leq u,\frac{T_{i}}{T_{i}+1}{\hat{F}}_{i}\left(X_{i(t+h)}\right)\leq v)\\ & = & \frac{1}{T_{i}-h}\sum_{t=1}^{T_{i}-h}I(F_{i}\left(X_{it}\right)\leq \frac{T_{i}+1}{T_{i}}u-\left({\hat{F}}_{i}\left(X_{it}\right)-F_{i}\left(X_{it}\right)\right))\cdot \\ & & I(F_{i}\left(X_{i(t+h)}\right)\leq \frac{T_{i}+1}{T_{i}}v-\left({\hat{F}}_{i}\left(X_{i(t+h)}\right)-F_{i}\left(X_{i(t+h)}\right)\right)) \end{array}$$
  On the event *A*_*i*_, for an arbitrary *ϵ*, there exists a *T* such that $|{\hat{F}}_{i}\left(x\right)-F_{i}\left(x\right)|<\epsilon$ for ∀*x* ∈ (−∞, ∞). Hence we have that $I(F_{i}\left(X_{it}\right)\leq \frac{T_{i}+1}{T_{i}}u-\epsilon )\leq I(F_{i}\left(X_{it}\right)\leq \frac{T_{i}+1}{T_{i}}u-\left({\hat{F}}_{i}\left(X_{it}\right)-F_{i}\left(X_{it}\right)\right))\leq I(F_{i}\left(X_{it}\right)\leq \frac{T_{i}+1}{T_{i}}u+\epsilon )$ and $I(F_{i}\left(X_{i(t+h)}\right)\leq \frac{T_{i}+1}{T_{i}}v-\epsilon )\leq I(F_{i}\left(X_{i(t+h)}\right)\leq \frac{T_{i}+1}{T_{i}}v-\left({\hat{F}}_{i}\left(X_{i(t+h)}\right)-F_{i}\left(X_{i(t+h)}\right)\right))\leq I(F_{i}\left(X_{i(t+h)}\right)\leq \frac{T_{i}+1}{T_{i}}v+\epsilon )$. Consequently,
  $$\begin{array}{c} \frac{1}{T_{i}-h}\sum_{t=1}^{T_{i}-h}I(F_{i}\left(X_{it}\right)\leq \frac{T_{i}+1}{T_{i}}u-\epsilon ,F_{i}\left(X_{i(t+h)}\right)\leq \frac{T_{i}+1}{T_{i}}v-\epsilon )\leq {\hat{C}}_{i,h}\left(u,v\right), \end{array}$$
  and
  $$\begin{array}{c} \frac{1}{T_{i}-h}\sum_{t=1}^{T_{i}-h}I(F_{i}\left(X_{it}\right)\leq \frac{T_{i}+1}{T_{i}}u+\epsilon ,F_{i}\left(X_{i(t+h)}\right)\leq \frac{T_{i}+1}{T_{i}}v+\epsilon )\geq {\hat{C}}_{i,h}\left(u,v\right). \end{array}$$
  This means that ${\tilde{C}}_{i,h}\left(\frac{T_{i}+1}{T_{i}}u-\epsilon ,\frac{T_{i}+1}{T_{i}}v-\epsilon \right)\leq {\hat{C}}_{i,h}\left(u,v\right)\leq {\tilde{C}}_{i,h}\left(\frac{T_{i}+1}{T_{i}}u+\epsilon ,\frac{T_{i}+1}{T_{i}}v+\epsilon \right)$. Then let *T*_*i*_ → ∞, by the result of Step 1, with probability 1 we have
  $$\begin{array}{c} C_{i,h}\left(u-\epsilon ,v-\epsilon \right)\leq {\underline{\lim}}_{T_{i}\to \infty }{\hat{C}}_{i,h}\left(u,v\right)\leq {\bar{\lim}}_{T_{i}\to \infty }{\hat{C}}_{i,h}\left(u,v\right)\leq C_{i,h}\left(u+\epsilon ,v+\epsilon \right). \end{array}$$
  Finally, by the arbitrary property of *ϵ*, one can obtain that as *T*_*i*_ → ∞, ${\hat{C}}_{i,h}\left(u,v\right)\overset{a.s.}{\to }C_{i,h}\left(u,v\right).$
Step 4 In this step, we will prove that ${\hat{D}}_{h}\left(i,i^{\prime }\right)\overset{a.s.}{\to }D_{h}\left(i,i^{\prime }\right)$ as *T*_*i*_, *T*_*i*′_ → ∞. By the results of Step 3, we have as *T*_*i*_, *T*_*i*′_ → ∞, ${\hat{C}}_{i,h}\left(u,v\right)\overset{a.s.}{\to }C_{i,h}\left(u,v\right)$ and ${\hat{C}}_{i^{\prime },h}\left(u,v\right)\overset{a.s.}{\to }C_{i^{\prime },h}\left(u,v\right)$, which means that ${\left({\hat{C}}_{i,h}\left(u,v\right)-{\hat{C}}_{i^{\prime },h}\left(u,v\right)\right)}^{2}\overset{a.s.}{\to }{\left(C_{i,h}\left(u,v\right)-C_{i^{\prime },h}\left(u,v\right)\right)}^{2}$. Moreover, ${\left({\hat{C}}_{i,h}\left(u,v\right)-{\hat{C}}_{i^{\prime },h}\left(u,v\right)\right)}^{2}\leq 1$, then by the dominated convergence theorem, one can obtain that ${\hat{D}}_{h}\left(i,i^{\prime }\right)\overset{a.s.}{\to }D_{h}\left(i,i^{\prime }\right)$ as *T*_*i*_, *T*_*i*′_ → ∞.

This completes the whole proof of Theorem 1.

### Appendix B: Proof of Proposition 1

By the Eq (2), we have

$$\begin{array}{c} {\hat{D}}_{h}\left(i,i^{\prime }\right)=\sqrt{\int \int_{{\left[0,1\right]}^{2}}({\hat{C}}_{i,h}{\left(u,v\right)}^{2}-2{\hat{C}}_{i,h}\left(u,v\right){\hat{C}}_{i^{\prime },h}\left(u,v\right)+{\hat{C}}_{i^{\prime },h}{\left(u,v\right)}^{2})dudv}. \end{array}$$

By the defination of ${\hat{C}}_{i,h}\left(u,v\right)$, one can see that

$$\begin{array}{ccc} & & \int \int_{{\left[0,1\right]}^{2}}{\hat{C}}_{i,h}{\left(u,v\right)}^{2}dudv\\ & = & \frac{1}{{\left(T_{i}-h\right)}^{2}}\int \int_{{\left[0,1\right]}^{2}}\sum_{t=1}^{T_{i}-h}\sum_{t^{\prime }=1}^{T_{i}-h}I\left(U_{it}\leq u\right)I\left(V_{it}\leq v\right)I\left(U_{it^{\prime }}\leq u\right)I\left(V_{it^{\prime }}\leq v\right)dudv\\ & = & \frac{1}{{\left(T_{i}-h\right)}^{2}}\sum_{t=1}^{T_{i}-h}\sum_{t^{\prime }=1}^{T_{i}-h}\int_{0}^{1}I\left(U_{it}\leq u\right)I\left(U_{it^{\prime }}\leq u\right)du\cdot \int_{0}^{1}I\left(V_{it}\leq v\right)I\left(V_{it^{\prime }}\leq v\right)dv\\ & = & \frac{1}{{\left(T_{i}-h\right)}^{2}}\sum_{t=1}^{T_{i}-h}\sum_{t^{\prime }=1}^{T_{i}-h}\left(1-\max\left(U_{it},U_{it^{\prime }}\right)\right)\left(1-\max\left(V_{it},V_{it^{\prime }}\right)\right)\\ & = & L_{i,i}. \end{array}$$

Similarly, one can also verify that $\int \int_{{\left[0,1\right]}^{2}}{\hat{C}}_{i,h}\left(u,v\right){\hat{C}}_{i^{\prime },h}\left(u,v\right)dudv=L_{i,i^{\prime }}$. Hence we have that ${\hat{D}}_{h}\left(i,i^{\prime }\right)=\sqrt{L_{i,i}-2L_{i,i^{\prime }}+L_{i^{\prime },i^{\prime }}}.$

This completes the proof of Proposition 1.

### Appendix C: Proof of Theorem 2

By the results of Theorem 1, we have that as min_*i*_{*T*_*i*_} → ∞, ${\hat{D}}_{h}\left(i,i^{\prime }\right)\overset{a.s.}{\to }D_{h}\left(i,i^{\prime }\right)$ for all *i*, *i*′ = 1, ⋯, *n*. Hence one can prove the Theorem on the event $\left\{{\hat{D}}_{h}\left(i,i^{\prime }\right)\to D_{h}\left(i,i^{\prime }\right)\;\text{for}\;\text{all}\;i,i^{\prime }=1,\cdots ,n\right\}$, which is denoted by E.

Assume that *X*_*i*_ and *X*_*i*′_ belong to the *j*-th cluster and the *j*′-th cluster respectively, then we have that *D*_*h*_(*i*, *i*′) = *D*_0,*h*_(*j*, *j*′). One can see that *D*_0,*h*_(*j*, *j*′) = 0 for *j* = *j*′, and *D*_0,*h*_(*j*, *j*′) ≥ *ϵ* for *j* ≠ *j*′. Consequently, on the event E, as long as min_*i*_{*T*_*i*_} is large enough we have ${\hat{D}}_{h}\left(i,i^{\prime }\right)<\epsilon /2$ if *X*_*i*_, *X*_*i*′_ belong to a common cluster, and ${\hat{D}}_{h}\left(i,i^{\prime }\right)>\epsilon /2$ if *X*_*i*_, *X*_*i*′_ belong to different clusters. Let $\left(i_{1},i_{1}^{\prime }\right)=\arg\min_{\left(i,i^{\prime }\right)}{\hat{D}}_{h}\left(i,i^{\prime }\right)$, then $X_{i_{1}},X_{i_{1}^{\prime }}$ must belong to the common cluster, and for *J* = *n* in the algorithm, $X_{i_{1}},X_{i_{1}^{\prime }}$ are merged together as a cluster. This means that for *J* = *n*, the theorem holds.

Next assume the theorem holds for *J* = *J*_1_ + 1 > *J*_0_ + 1, we will prove the theorem still holds for *J* = *J*_1_. For *J* = *J*_1_ + 1, we denote the *J*_1_ clusters as ${\hat{M}}_{1},\cdots ,{\hat{M}}_{J_{1}}$. Here one should define the dissimilarities among clusters. Without loss of generality, we only consider the single linkage method here and the proof for other dissimilarities among clusters is similar. For single linkage, the distance between ${\hat{M}}_{j},{\hat{M}}_{j^{\prime }}$ is defined as $\hat{D}\left({\hat{M}}_{j},{\hat{M}}_{j^{\prime }}\right)=\min_{X_{i}\in {\hat{M}}_{j},X_{i^{\prime }}\in {\hat{M}}_{j^{\prime }}}\hat{D}\left(i,i^{\prime }\right)$. Based on the assumption that the theorem holds for *J* = *J*_1_ + 1 > *J*_0_, we know that time series in each ${\hat{M}}_{j}$ share a common copula function. Then we have that $\hat{D}\left({\hat{M}}_{j},{\hat{M}}_{j^{\prime }}\right)<\epsilon /2$ if ${\hat{M}}_{i},{\hat{M}}_{j}$ belong a common cluster, and $\hat{D}\left({\hat{M}}_{j},{\hat{M}}_{j^{\prime }}\right)>\epsilon /2$ otherwise. Furthermore, due to *J*_1_ > *J*_0_ there exist at least two ${\hat{M}}_{j},{\hat{M}}_{j^{\prime }}$, time series in which also share a common coplua function. Let $\left(\hat{j},{\hat{j}}^{\prime }\right)=\arg\min_{j\neq j^{\prime }}\hat{D}\left({\hat{M}}_{j},{\hat{M}}_{j^{\prime }}\right)$. For *J* = *J*_1_, ${\hat{M}}_{\hat{j}}$ and ${\hat{M}}_{{\hat{j}}^{\prime }}$ will be merged togother as a new cluster. Moreover, by the above analysis, we know ${\hat{M}}_{\hat{j}}$ and ${\hat{M}}_{{\hat{j}}^{\prime }}$ share a common copula function, and they are truely belong to a common cluster. This means that the claims of Theoren 2 is ture for *J* = *J*_1_.

This completes the whole proof of Theorem 2.

## References

[1] Frühwirth-Schnatter S, Kaufmann S. Model-based clustering of multiple time series. Journal of Business & Economic Statistics. 2008;26(1):78–89. 10.1198/073500107000000106

[2] Xiong Y, Yeung DY. Time series clustering with ARMA mixtures. Pattern Recognition. 2004;37(8):1675–1689. 10.1016/j.patcog.2003.12.018

[3] Otranto E. Clustering heteroskedastic time series by model-based procedures. Computational Statistics & Data Analysis. 2008;52(10):4685–4698. 10.1016/j.csda.2008.03.020

[4] Ramoni M, Sebastiani P, Cohen P. Bayesian Clustering by Dynamics. Machine Learning. 2002;47(1):91–121. 10.1023/A:1013635829250

[5] Oates T, Firoiu L, Cohen PR. Clustering Time Series with Hidden Markov Models and Dynamic Time Warping. In Proceedings of the IJCAI-99 Workshop on Neural, Symbolic and Reinforcement Learning Methods for Sequence Learning; 1999.

[6] Montero P, Vilar JA. TSclust: An R package for time series clustering. Journal of Statistical Software. 2014;62(1):1–43. doi: 10.18637/jss.v062.i01

[7] Liao TW. Clustering of time series data: a survey. Pattern Recognition. 2005;38(11):1857–1874. 10.1016/j.patcog.2005.01.025

[8] Piccolo D. A distance measure for classifying ARIMA models. Journal of Time Series Analysis. 1990;11(2):153–164. 10.1111/j.1467-9892.1990.tb00048.x

[9] Maharaj EA. Cluster of time series. Journal of Classification. 2000;17(2):297–314. 10.1007/s003570000023

[10] Kalpakis K, Gada D, Puttagunta V. Distance measures for effective clustering of ARIMA time-series. In: Data Mining, 2001. ICDM 2001, Proceedings IEEE International Conference on. IEEE; 2001. p. 273–280.

[11] Corduas M, Piccolo D. Time series clustering and classification by the autoregressive metric. Computational Statistics & Data Analysis. 2008;52(4):1860–1872. 10.1016/j.csda.2007.06.001

[12] Liu S, Maharaj EA. A hypothesis test using bias-adjusted AR estimators for classifying time series in small samples. Computational Statistics & Data Analysis. 2013;60:32–49. 10.1016/j.csda.2012.11.014

[13] Galeano P, Peña DP. Multivariate Analysis in Vector Time Series. Resenhas. 2000;4:383–404.

[14] D’Urso P, Maharaj EA. Autocorrelation-based fuzzy clustering of time series. Fuzzy Sets and Systems. 2009;160(24):3565–3589. 10.1016/j.fss.2009.04.013

[15] Caiado J, Crato N, Peña D. A periodogram-based metric for time series classification. Computational Statistics & Data Analysis. 2006;50(10):2668–2684. 10.1016/j.csda.2005.04.012

[16] Bohte Z, Cepar D, Kosmelj K. Clustering of time series. In: Compstat. vol. 80; 1980. p. 587–593.

[17] Díaz SP, Vilar JA. Comparing several parametric and nonparametric approaches to time series clustering: A simulation study. Journal of classification. 2010;27(3):333–362. 10.1007/s00357-010-9064-6

[18] Rémillard B, Scaillet O. Testing for equality between two copulas. Journal of Multivariate Analysis. 2009;100(3):377–386. 10.1016/j.jmva.2008.05.004

[19] Sklar M. Fonctions de répartition à n dimensions et leurs marges. Publ inst statist univ Paris. 1959;8:229–231.

[20] Lafuente-Rego B, Vilar J. Clustering of time series using quantile autocovariances. Advances in Data Analysis & Classification. 2016;10(3):391–415. 10.1007/s11634-015-0208-8

[21] Kaufman L, Rousseeuw PJ. Finding groups in data: an introduction to cluster analysis. John Wiley & Sons; 2009.

[22] Luxburg U. A tutorial on spectral clustering. Statistics & Computing. 2007;17(4):395–416. 10.1007/s11222-007-9033-z

[23] Hastie T, Tibshirani R, Friedman J. The elements of statistical learning 2nd edition New York: Springer; 2009.

[24] James G, Witten D, Hastie T, Tibshirani R. An introduction to statistical learning with Applications in R. Springer; 2013.

[25] Lance GN, Williams WT. A General Theory of Classificatory Sorting Strategies. Hierarchical systems. The Computer Journal. 1967;9(4):373–380. 10.1093/comjnl/9.4.373

[26] Batagelj V. Generalized Ward and Related Clustering Problems. In: Hh Bock, Classification & Related Methods of Data Analysis; 1988. p. 67–74.

[27] Rousseeuw PJ. Silhouettes: a graphical aid to the interpretation and validation of cluster analysis. Journal of computational and applied mathematics. 1987;20:53–65. 10.1016/0377-0427(87)90125-7

[28] Maharaj EA. A significance test for classifying ARMA models. Journal of Statistical Computation and Simulation. 1996;54(4):305–331. 10.1080/00949659608811737

[29] Gavrilov M, Anguelov D, Indyk P, Motwani R. Mining the stock market (extended abstract): which measure is best? In: Proceedings of the sixth ACM SIGKDD international conference on Knowledge discovery and data mining. ACM; 2000. p. 487–496.

[30] Tong H, Yeung I. On Tests for Self-Exciting Threshold Autoregressive-type Nonlinearity in Partially Observed Time-Series. Applied Statistics-Journal of the Royal Statistical Society Series C. 1991;40(1):43–62.

[31] Petruccelli JD, Woolford S. A Threshold AR(1) Model. Journal of Applied Probability. 1984;21(02):270–286. 10.1017/S0021900200024670

[32] Chan KS, Petruccelli JD, Tong H, Woolford S. A Multiple-Threshold AR(1) Model. Journal of Applied Probability. 1985;22(02):267–279. 10.2307/3213771

[33] Chan KS, Tsay RS. Limiting properties of the least squares estimator of a continuous threshold autoregressive model. Biometrika. 1998;85(2):413–426. 10.1093/biomet/85.2.413

[34] Liu W, Ling S, Shao Q. On non-stationary threshold autoregressive models. Bernoulli. 2011;17(3):969–986. 10.3150/10-BEJ306

[35] Borg I, Groenen PJ. Modern multidimensional scaling: Theory and applications. Springer Science & Business Media; 2005.

[36] Atkinson AB, Bourguignon F. Handbook of Income Distribution. Elvesier; 2000.

[37] Fan J. Nonlinear time series: nonparametric and parametric methods. Springer; 2003.

[38] Tucker HG. A Generalization of the Glivenko-Cantelli Theorem. Annals of Mathematical Statistics. 1959;30(3):828–830. 10.1214/aoms/1177706212

